# Editorial: Frailty and Herbal Medicines- From Molecular Mechanisms to Clinical Efficacy

**DOI:** 10.3389/fnut.2020.00041

**Published:** 2020-04-15

**Authors:** Koji Ataka, Ryuji Takahashi, Jiang Bo Li, Norio Iizuka, Akio Inui

**Affiliations:** ^1^Pharmacological Department of Herbal Medicine, Kagoshima University Graduate School of Medicine and Dental Sciences, Kagoshima, Japan; ^2^Kampo Research Laboratories, Kracie Pharma, Ltd., Tokyo, Japan; ^3^Department of Clinical Psychology, Second People's Hospital of Wuhu, Wuhu, China; ^4^Department of Kampo Medicine, Graduate School of Biomedical & Health Sciences, Hiroshima University, Hiroshima, Japan

**Keywords:** herbal medicine, Kampo medicine, Ninjin'yoeito, frailty, aging

In East Asia, natural foods have been used as medicine and have played important roles in human health for centuries, based on the concept of “Eating healthy prevents and cures the disease” in Traditional Chinese medicine. Herbal medicines consist of various natural components obtained from the leaves, roots, and seeds of plants, combined with products from animal, fungal, and mineral origin. The use of herbal medicine has been recently gaining global interest (1). The World Health Organization (WHO) has proposed several guidelines for the use of herbal medicine (2–9), and the Traditional Medicine Strategy 2014–2023 is being undertaken (10). The WHO encourages the integration of traditional medicine into the next edition of the International Statistical Classification of Diseases and Related Health Problems (International Statistical Classification of Diseases and Related Health Problems-11, ICD-11) (11). However, herbal medicines are not fully accepted in clinical practice, and more evidence is required regarding the appropriate and safe usage of herbal medicines to be accepted as mainstream medicines. Traditional Chinese medicine has been part of human history for several thousands of years. The earliest extant medical book is called Huangdi Neijing (Canon of Internal Medicine, 770 BC~221 BC), while the earliest pharmacy monograph is known to be the Sheng Nong Bencao Jing (Sheng Nong Materia Medica, AD25~AD220) (12). Kampo medicine originates from Traditional Chinese medicine, while it was developed in Japan (13).

Herbal medicines in both Traditional Chinese medicine and Kampo medicine have been reported to have significantly beneficial effects, including fatigue- and pain-relief, reduction of infection in the respiratory tract, reduction of diarrhea, nausea, and vomiting, protection of the liver, as well as amelioration of the symptoms of cachexia. Recently, the number of clinical studies associated with frailty has increased; however, effective treatments have been only sporadically reported. Conversely, treatments using herbal medicines for frailty have been extensively published in Japanese journals, although such knowledge has not yet become global.

Frailty is characterized by unintentional weight loss, exhaustion, muscle weakness, slow walking speed, and low physical activity in elderly people, and is associated with an increased vulnerability to stressors and impairment of cognitive and emotional functions. Furthermore, various chronic diseases such as cancer, diabetes, respiratory disease, and psychosomatic disease worsen with the progress of frailty. These disorders associated with frailty have a significant rate of success when they are treated with Kampo medicine (Takayama et al.).

Kampo medicine is composed of various herbal products and is effective against a wide range of diseases. In Japan herbal medicine is prescribed following the theory of Kampo medicine, which is completely different from that of Western medicine. These differences of theory, therefore, make the performance of clinical studies in Western countries difficult. Researchers, who perform experiments using Kampo medicine, should continue providing evidence that Kampo medicine is effective against frailty and aging in order to spread the knowledge and facilitateits worldwide approval.

This Research Topic titled “Frailty and Herbal Medicines—From Molecular Mechanisms to Clinical Efficacy” was planned to provide scientific evidence focused on the mechanisms of herbal medicines in basic experiments. In this Research Topic, 30 articles are presented: 17 basic research studies; three clinical research studies, seven reviews; and three other articles (Tables 1–4). Various herbal medicines have been suggested as useful therapeutic options for frailty and frailty-associated symptoms. In the Kampo theory doctors and pharmacists analyze the syndromes and signs as well as the patient's history, and then the appropriate herbal medicine is selected. These syndromes are called “Sho” in the Kampo theory, which is categorized as three different indicators: Qi (well-being, energy, illness, and vigor), Blood, and Water (14). The arising abnormalities of Qi, Blood, and Water are considered the causes of diseases. The source of Qi is called “Jin.” The loss of Jin, “Jinkyo,” induces a deficiency of psychological and physical energy, which influences growth and reproductive functions in human beings. Jinkyo causes various symptoms such as loss of hair, gray hair, weariness, lumbago, osteoporosis, incontinence, cold legs, itching, and difficulty in hearing. These are all considered to be causes of frailty and aging in the Kampo theory. Hozai is an herbal medicine used to improve the loss of Qi and Blood. Ninjin'yoeito (NYT) is a Hozai formulation in Kampo medicine. NYT is composed of 12 crude drugs (Rehmannia root, Japanese angelica root, *Atractylodes* rhizome, *Poria* sclerotium, ginseng, cinnamon bark, polygala root, peony root, citrus unshiu peel, astragalus root, *Glycyrrhiza*, and *Schisandra* fruit). NYT has been proven effective against weakness, general malaise, fatigue, anorexia, night sweats, coldness, and anemia (ICD-10). These symptoms fall into categories such as the loss of Qi and Blood. NYT has been reported to include various active components, which have been reviewed by Uto et al. in this Research Topic. The efficacy and safety of NYT are also summarized in this topic. Similarly, the effects of many herbal medicines and their components except for Hozai are reported. Endogenous stem cells such as bone marrow, adipose cells, and umbilical cord-derived mesenchymal stem cells have multifactorial functions and secrete various factors. These characteristics may be similar to those of herbal medicines containing various active ingredients. The potential of mesenchymal stem cells for the therapy of frailty is described in this topic. A combination of stem cell therapy and herbal medicine may work well for the treatment of frailty.

**Table 1:** Herbal medicines in basic studies of present Research Topics.

**Name**	**Models**	
Lumbrokinase from *Lumbricus rubellusn*	Myocardial ischemia-reperfusion injury in rats	Wang Y.-H. et al.
Li-Ru-Kang	Hyperplasia of mammary glands in rats	Wei et al.
Jinshui Huanxian formula	Pulmonary fibrosis in rats	Bai et al.
Aspidinol from *Dryopteris fragrans*	Methicillin-resistant *Staphylococcus aureus*	Hua et al.
Total saponins from *Phytolacca acinosa* and *P. Americana*	Hyperplasia of mammary glands in rats	Li et al.
*Gardenia jasminoides Ellis*	Postmenopausal syndrome model using primary ovarian granulosa cells from rats	Wang X. et al.
Notoginsenoside Fc from *Panax notoginseng*	Platelets in rats	Liu et al.
Total glycoside from *Rehmannia glutinosa*	Diabetic nephropathy in rats	Dai et al.
Hangkui capsule from *Abelmoschus manihot* (L.) medic	Diabetic nephropathy in rats	Wu et al.
Ninjin'yoeito	Skeketal muscle function in cancer-bearing mice	Ohsawa et al.
Ninjin'yoeito (Ninjinyoeito)	Depression induced by corticosterone in mice	Murata et al.
Ninjin'yoeito	Diabetes mellitus symptoms	Hosogi et al.
Extract from Maca (*Lepidium meyenii* Walpers)	High-fat, high-fructose diet-induced metabolism disorder in golden hamsters	Wan et al.
Semen Cassiae extract from *Cassia obtusifolia* or *C. tora* L.	Glucose metabolism in skeletal muscle of diabetic rats	Zhang et al.
Isoliquiritigenin from Licorice (*Glycyrrhiza uralensis*)	Hepatotoxicity induced by triptolide from T*ripterygium wilfordii* Hook E. in human hepatocytes	Hou et al.
Tricaproin from *Simarouba glauca*	Colorectal carcinoma cell lines	Jose et al.
Ciji-Hua'ai-Basosheg II Formula	Chemotherapy of mice with transplanted H_22_ hepatocellular carcinoma	Fu et al.

**Table 2:** Herbal medicines in clinical studies of present Research Topics.

**Name**	**Diseases**	
Ninjin'yoeito	Elderly patients (post-marketing survey)	Suzuki et al.
Ninjin'yoeito	Frail	Sakisaka et al.
Ninjin'yoeito	Fatigue in patients with lenalidomide and dexamethasone	Ito et al.

**Table 3:** Reviews of present Research Topics.

**Name**	**Diseases**	
*Panax ginseng*	Frailty-related disorders	Ogawa-Ochiai and Kawasaki
Ninjin'yoeito	Elderly patients (post-marketing survey)	Suzuki et al.
Ninjin'yoeito	Sarcopenia and frailty	Uto et al.
Ninjin'yoeito (Ninjinyoueito)	Various diseases and symptoms	Miyano et al.
Renshen Yangrong Decoction	Asthenic disease symptoms	Li et al.
Kampo	Frailty in locomotor disease	Nakae et al.
Mesenchymal stem cells	Aging frailty	Schulman et al.

**Table 4:** Other in present Research Topics.

**Subject**	**Target**	
Association between appetite and sarcopenia	Mild cognitive impairment and early-stage Alzheimer's disease	Kimura et al.
Clinical practice guidelines for Kampo	Frailty	Takayama et al.
*Caenorhabditis elegans*	Animal model for screening drugs	Matsunami

We hope that this Research Topic on the potential of herbal medicines will be informative. We believe that herbal medicines can become a mainstream treatment for frailty within a considerable period.

## Author Contributions

The manuscript is written by KA. AI conceived and organized the structure of the editorial. AI, RT, JL, and NI contributed to the critical revision and approved the final manuscript for publication.

### Conflict of Interest

KA and AI work in a laboratory funded by Kracie. RT works in the laboratory of Kracie. The remaining authors declare that the research was conducted in the absence of any commercial or financial relationships that could be construed as a potential conflict of interest.
